# Clinical practice guideline for drug-induced kidney injury in Japan 2016: digest version

**DOI:** 10.1007/s10157-016-1334-0

**Published:** 2016-10-06

**Authors:** Joichi Usui, Kunihiro Yamagata, Eri Imai, Hiroshi Okuyama, Hiroshi Kajiyama, Hiroshi Kanamori, Shuzo Kaneko, Emiko Kono, Yukinao Sakai, Norihiko Sakai, Yuichi Sakamaki, Yoshinori Taniguchi, Kentaro Nakai, Hiroki Nishiwaki, Sumio Hirata, Hideki Yamaya, Shuichi Tsuruoka, Yoshio Terada, Hitoshi Yokoyama, Takashi Wada, Ichiei Narita

**Affiliations:** 1 Department of Nephrology, Faculty of Medicine, University of Tsukuba, Tsukuba, Ibaraki Japan; 2 Department of Nephrology, Itabashi Chuo Medical Center, Tokyo, Japan; 3 Department of Nephrology, Asanogawa General Hospital, Kanazawa, Japan; 4 Department of Rheumatology and Applied Immunology, Faculty of Medicine, Saitama Medical University, Moroyama-chou, Saitama, Japan; 5 Department of Nephrology, Fukuchiyama City Hospital, Fukuchiyama, Japan; 6 Department of Nephrology, Nagaoka Chuo General Hospital, Nagaoka, Japan; 7 Department of Nephrology, Graduate School of Medicine, Nippon Medical School, Tokyo, Japan; 8 Division of Blood Purification and Nephrology, Kanazawa University Hospital, Kanazawa, Japan; 9 Clinical Nephroscience, Niigata University Graduate School of Medical and Dental Sciences, Niigata, Japan; 10 Division of Clinical Nephrology and Rheumatology, Niigata University Graduate School of Medical and Dental Sciences, Niigata, Japan; 11 Department of Endocrinology, Metabolism and Nephrology, Kochi Medical School, Kochi University, Nankokum, Kochi Japan; 12 Division of Nephrology and Kidney Center, Kobe University Graduate School of Medicine, Kobe, Japan; 13 Center for Innovative Research for Communities and Clinical Excellence, Fukushima Medical University, Fukushima, Japan; 14 Division of Clinical Pharmacology, Center for Clinical Pharmaceutical Sciences, Faculty of Pharmaceutical Sciences, Kumamoto University, Kumamoto, Japan; 15 Department of Nephrology, Kanazawa Medical University School of Medicine, Uchinada, Kanazawa, Japan; 16 Division of Nephrology, Department of Laboratory Medicine, Kanazawa University, Kanazawa, Japan

## Introduction

Approximately one in eight adults has chronic kidney disease (CKD) in Japan, and the prevalence rate is expected to rise steeply due to the aging of the population in this country. In patients with CKD, quite a few medications require the dosage reduction or discontinuation because of their reduced urinary excretion and the increased risk of further renal impairment. Therefore, CKD patients are often treated by insufficient amounts of the medications, even though they may suffer from various complications. Moreover, it is empirically known that drug-induced kidney injury (DKI) accelerates the progression of renal failure, while it is not superficially ranked as a primary cause of kidney disease.

In this context, the early detection, prevention, and treatment of DKI are very important issue in preventing the progression of CKD and the development of renal failure. However, there are no comprehensive and practical guideline on the diagnosis and treatment of DKI for CKD patients and on dosage adjustments for these patients.

In response to this need, a clinical practice guideline for DKI was developed with the support of a Health and Labour Science Research Grant from the Ministry of Health, Labour, and Welfare (MHLW) and the Japan Agency for Medical Research and Development (AMED) for Practical Research Project for Renal Diseases, “Early detection and treatment of drug-induced kidney injury that aggravate chronic kidney disease.” This guideline was established by doing a clinical survey on DKIs, evaluating clinicopathological factors, investigating the methods of the early detection of the disease, and analyzing animal models. The present article represents a Committee of Clinical Practice Guideline for DKI. We collected supportive evidence and analyzed data, focusing on several clinical questions that have practical importance.

## Evidence levels, total evidence grades and recommendation levels

The level of each evidence was determined according to the method of previous Japanese Clinical Practice Guidelines, namely, as using the abridged English version of evidence-based clinical practice guidelines for CKD [1]. In brief, evidence was classified into six levels based on the study design, and was arranged roughly from the most reliable study type (Level 1) to the least reliable (Level 6). These levels do not necessarily represent rigorous scientific standards. As a result, total evidence grades for each clinical question were determined based on the evidence level for each question. Finally, the members of the committee discussed the matter and decided on the recommendation levels based on the total evidence grades or expert consensus.

### Evidence levels

Level 1: systematic review/meta-analysis.

Level 2: at least one randomized controlled trial (RCT).

Level 3: a non-randomized controlled trial.

Level 4: an analytical epidemiologic study (cohort study or case–control study) or a single-arm intervention study (no controls).

Level 5: a descriptive study (case report or case series).

Level 6: opinion of an expert committee or an individual expert, which is not based on patient data.

### Total evidence grades

Grade A (strong): the scientific basis is strong.

Grade B (moderate): the scientific basis is moderate.

Grade C (weak): the scientific basis is limited.

Grade D (very weak): there is no scientific basis.

### Recommendation levels

Level 1: strongly recommended.

Level 2: weakly recommended or suggested.

Level undefined: without recommendation.

## Section 1. Definition, classification, and practice of DKI

The definition of DKI is a new onset of kidney injury or the worsening of an existing kidney injury due to drug administration. DKI can be classified based on the mechanism of pathogenesis, as well as on the damaged segment of the kidney. The classification based on the mechanism of pathogenesis is as follows: (1) toxic kidney injury (direct toxicity); (2) acute interstitial nephritis (AIN) due to allergic mechanism (hypersensitivity and direct toxicity); (3) indirect toxicity, such as electrolyte abnormalities and decrease of renal blood flow; and (4) obstruction of urinary tract. The classification based on the damaged segment of kidney is as follows: (1) glomerular injury; (2) tubular injury; (3) interstitial injury; and (4) vascular injury. The criteria for diagnosis are as follows: (1) new onset of kidney injury after the start of the administration of the candidate agent and (2) improvement or stoppage of the progression of the kidney injury after the cessation of the candidate agent, and all other causes can be ruled out. The cornerstone of treatment is the identification and cessation of the candidate agent as soon as possible.

## Question 1. Is eosinouria a useful biomarker for the early diagnosis of DKI?

### Statements

1. Eosinouria can be detected in DKI due to AIN, but it is not a useful biomarker for the diagnosis of DKI because of its high rate of false negatives (recommendation: Level 2; total evidence: Grade C).
2. If eosinouria is detected in acute kidney injury (AKI), acute tubular necrosis (ATN) can be excluded as a cause of the AKI (recommendation: Level 2; total evidence: Grade C).

## Question 2. Is a renal biopsy useful for the diagnosis of DKI?

### Statements

1. Renal biopsy is a useful tool for predicting the renal outcome of DKI and determining further treatment strategies (recommendation: Level 2; total evidence: Grade C).
2. Renal biopsy is a useful tool for the differential diagnosis between drug-induced renal tubular or interstitial injuries and other causes (recommendation: Level 2; total evidence: Grade C).
3. By renal biopsy, the glomerular histology can be confirmed and a valuable information on whether or not to suspect DKI can be available (recommendation: Level undefined; total evidence: Grade C).

## Section 2. Overview of the epidemiology of DKI in Japan

To clarify the epidemiology of DKI in Japan, we reviewed the reports of DKI for elderly persons from the study of a Health and Labour Science Research Grant from the Ministry of Health, Labour, and Welfare (2007–2009) and analyzed the data of the Japan-Kidney Disease Registry (J-KDR) from 2007 to 2012.

In the reports of DKI for elderly persons (2007–2009), DKI accounted for approximately 1 % of all admitted patients in the hospitals of 47 representative nephrologists. The major drugs inducing renal injury were non-steroidal anti-inflammatory drugs (NSAIDs) in 25.1 % of cases, anti-cancerous drugs in 18.0 %,antibiotic agents in 17.5 %, and radio-contrast agents in 5.7 %. Of these cases, 54.6 % were of the direct renal injury type. Moreover, the kidney function of 36.5 % of these patients did not recover.

A total of 231 cases of DKI, including 224 renal-biopsy-proven cases, had been registered on J-KDR in 2007–2012 (1.42 % of 15,821 cases). The frequency of DKI increased with aging. Elderly patients in their 70s showed a three times higher frequency of DKI as compared to less than 10 year (1.83 vs. 0.65 %). The major clinical diagnoses of these cases were DKI in 118 cases (51.1 %), nephrotic syndrome in 42 cases (18.2 %), chronic nephritic syndrome in 42 cases (17.7 %), and rapidly progressive nephritic syndrome in 19 cases (8.2 %). The pathological findings of these cases were glomerular injuries in 67 cases (29.0 %), acute tubule-interstitial injuries in 60 cases (26.0 %), chronic tubule-interstitial injuries in 55 cases (23.8 %), and sclerotic glomerular lesion and/or nephrosclerosis in 18 cases (7.8 %). Both acute and chronic tubulo-interstitial injuries were mainly related to the clinical diagnosis of DKI. On the other hand, nephrotic syndrome mainly due to membranous nephropathy was the major cause of glomerular injuries in 44.4 %. The prevalence of these diagnosis was peaked in the 60s and 70s in all categories, excepting for chronic tubulo-interstitial injuries, which peaked in their 30s and 40s. According to the risk category of CKD (heat map), 80.6, 75.9, and 40.9 % of acute and chronic tubulo-interstitial injuries and glomerular injury were categorized as high-risk (red zone) cases, respectively.

The causative drugs identified in 71 cases, which included bucillamine in 26 cases with membranous nephropathy, gemucitabine in 3 cases with thrombotic microangiopathy, and propyl thiouracil in 3 cases with ANCA-related nephritis.

## Section 3. Treatment of DKI

Prompt treatment based on the mechanisms of DKI is important to achieve recovery in renal function. DKI can be divided into four major categories as follows: dose-dependent direct renal drug toxicity, dose-independent renal drug toxicity associated with immunological reactions, indirect renal toxicity caused by decreased renal blood flow and electrolyte disorders, and intratubular precipitation of drug crystals with low solubility (see “Introduction”). The primary treatment is the discontinuation of the presumed causative drugs in all the cases. Steroid therapy may be considered when renal dysfunction remains even after the withdrawal of the presumed causative drug.

## Question 3. Is treatment with steroids better than that without steroids to improve renal function in patients with drug-induced AIN?

### Statements

1. Steroid therapy may be considered when renal dysfunction remains even after the withdrawal of the presumed causative drug (recommendation: Level 2; total evidence: Grade C).

## Section 4. Medication for patients with reduced kidney function

A change of the drug regimen(s) (such as a reduction of the dosage or increase of the dosing interval) should be considered when using drugs excreted by kidneys in patients with renal impairment. Therefore, precise evaluation of renal function by the estimation of the glomerular filtration rate (eGFR) is important before the prescription. Several equations for evaluating renal function have been developed; however, careful consideration is needed, because these equations also have limitations for their usage.

## Question 4. Is intrinsic creatinine clearance (CCr) more suitable than eGFR equation as an appropriate evaluation of the renal function for the adjustment of the drug dosage?

### Statements

1. The utility of eGFR is variable among clinical conditions. It is preferable to use intrinsic CCr in patients, whose muscle mass is decreasing, such as those with sarcopenia and bony body patients (recommendation: Level 2; total evidence: Grade C).
2. Cystatin C-based GFR can be used in patients, whose muscle mass is decreasing (recommendation: Level 2; total evidence: Grade C).
3. The eGFR equation for the adjustment of the drug dosage is limited in its accuracy (recommendation: Level 2; total evidence: Grade C).

## Section 5. Analgesic-related kidney injury

NSAIDs and acetaminophen are major analgesics. NSAID-related kidney injuries usually involve ischemic damage due to the inhibition of cyclooxygenase, and in clinical settings, NSAID-related kidney injuries often present as acute kidney injuries. As other types of NSAID-related kidney injuries, AIN and interstitial nephritis complicated with nephrotic syndrome occur rarely. By contrast, acetaminophen abuse is known to result in chronic renal failure due to renal papillary necrosis, calcinosis, and chronic interstitial nephritis. The primary treatment for all analgesic-related kidney injuries is the discontinuation of the causative medication.

## Question 5. Do selective cyclooxigenase-2 (COX-2) inhibitors cause fewer kidney injuries than nonselective COX inhibitors?

### Statements

1. NSAIDs, including selective COX-2 inhibitors, may cause acute kidney injuries. The occurrence rate of acute kidney injuries induced by selective COX-2 inhibitors and nonselective COX inhibitors showed no significant difference (recommendation: Level 2; total evidence: Grade A).
2. The occurrence rate of kidney dysfunction due to long-term usage showed no significant difference between selective COX-2 inhibitors and nonselective COX inhibitors (recommendation: Level 2; total evidence: Grade B).

## Section 6. Antimicrobial-agent-related kidney injury

The frequency of antimicrobial-agent-related kidney injury has recently increased because of the increase in the numbers of elderly patients and CKD patients. In particular, aminoglycoside antibiotics and glycopeptide-based drugs (vancomycin) need to be used carefully in CKD patients. In the administration of antibiotics to CKD patients, careful consideration of dose adjustments and dosing intervals is necessary, depending on renal function. Renal injury due to aminoglycoside antibiotics and vancomycin is considered to be concentration dependent and has been known to be associated with the trough level. Therapeutic drug monitoring (TDM) of drug levels and microbial sensitivities is generally the best guide to the use of antimicrobial agents in patients with impaired drug excretion.

## Question 6. Does TDM cause fewer kidney injuries than non-monitoring in the use of vancomycin for methicillin-resistant staphylococcus aureus (MRSA)-infected patients?

### Statements

1. Regular TDM can prevent kidney injuries due to the use of vancomycin for MRSA-infected patients (recommendation: Level 1; total evidence: Grade A).
2. Regular TDM can significantly secure clinical efficacy in the use of vancomycin for MRSA-infected patients (recommendation: Level 1; total evidence: Grade A).
3. Trough-level monitoring of vancomycin is useful in patients with unstable renal function or who require long therapeutic periods (recommendation: Level 1; total evidence: Grade B).

## Section 7. Immunosuppressive drugs for nephritic and/or nephrotic syndrome

With regard to immunosuppressive drugs used in the treatment of nephritic and/or nephrotic syndrome, renal dysfunction may have influence on the effects of these drugs that originally have nephrotoxicity. It should be considered that these medications sometimes cause DKI, especially in those with urinary tract excretion. In addition, elderly patients often have higher risk for accelerating DKI.

## Question 7. Does dose adjustment of cyclosporine by regular TDM lead to less nephrotoxicity in patients with nephrotic syndrome?

### Statements

1. Regular TDM can prevent proximal tubular injury due to the acute toxicity of cyclosporine (recommendation: Level 1; total evidence: Grade A).
2. Regular TDM can prevent microangiopathy and interstitial lesions (striate tubulo-interstitial fibrosis) due to the chronic toxicity of cyclosporine (recommendation: Level 2; total evidence: Grade B).
3. Potential nephrotoxicity due to the long-term cyclosporine use is histologically assessed by renal biopsy, if necessary (recommendation: Level 2; total evidence: Grade B).
